# The effects of a task shifted multi-component mental health intervention to support prosthetic and orthotic service users in Cambodia: a non-randomised controlled study

**DOI:** 10.1186/s13033-025-00681-x

**Published:** 2025-08-21

**Authors:** Alan Maddock, Thearith Heang, Nil Ean, Sisary Kheng, Paul Best, Nerrolyn Ramstrand

**Affiliations:** 1 Department of Health Psychology, School of Population Health, Royal College of Surgeons in Ireland, Dublin, Ireland; 2 Exceed Worldwide, Phnom Penh, Cambodia; 3 Department of Prosthetics and Orthotics, National Institute of Social Affairs, Phnom Penh, Cambodia; 4 The Center for Trauma Care and Research Organization (CTRO), Phnom Penh, Cambodia; 5 Department of Psychology, The Royal University of Phnom Penh, Phnom Penh, Cambodia; 6 Centre for Technological Innovation, Mental Health and Education (TIME), Queens University Belfast, Belfast, Northern Ireland; 7 Department of Rehabilitation, School of Health and Welfare, Jönköping University, Box 1026, Jönköping, 551 11 Sweden

## Abstract

**Background:**

Mental health disorders are major public health challenges, particularly in low- and middle-income countries such as Cambodia, where chronic shortages in mental health services and human resources exist. These issues are compounded for marginalized groups such as persons with physical disabilities due to their being at a higher risk of psychological distress and PTSD symptoms. The development of effective and accessible mental health systems in Cambodia will require evidence-based culturally appropriate mental health interventions. There are insufficient good-quality studies which have been completed to draw firm conclusions about the effectiveness of culturally appropriate mental health interventions in Cambodia. The aim of this study was to evaluate the effectiveness of a task shifted multi-component mental health intervention (named ‘Friendship groups’) at reducing psychological distress and PTSD, worry, rumination and increasing mindfulness among cohort of prosthetic and orthotic (P&O) service users.

**Methods:**

From March 2023 to June 2024 data (*N* = 465) were collected as part of a mental health screening programme for P&O service users across Cambodia. Participants experiencing mild to moderate psychological distress were screened and invited to participate in this study. Due to ethical considerations a non-randomised controlled trial design with repeated measures (pre-post intervention) was conducted to evaluate the effects of Friendship groups (*N* = 33) against a non-active control condition (*N* = 39).

**Results:**

When compared to the control condition the Friendship groups were found to have a moderate to large effect on psychological distress, and a small to moderate effect on rumination. The Friendship group participants experienced reduced PTSD symptoms, worry and improved mindfulness post group but these changes were not significant when compared to the control condition.

**Conclusions:**

The results from this study evidence the acceptability and effectiveness of Friendship groups at reducing psychological distress. Our results also provide clear guidance that if Friendship groups are implemented across P&O services in Cambodia, P&O service users are likely to experience reduced psychological distress. These findings also add to the growing literature supporting the need for culturally adapted task shifted mental health interventions in low- and middle-income countries, where access to specialised care remains limited.

## Introduction

The prevalence of mental health disorders is markedly higher in low-to-middle income (LMIC) countries than in high income countries [1]. Research which has examined the prevalence of common mental health disorders in Cambodia have reported very high rates of depression (16.7%), anxiety (27.4%) and PTSD (7.6%) [2]. There are a number of factors which may help to explain the high rates of common mental health disorders amongst Cambodians. The Khmer Rouge genocide, which still casts a long intergenerational shadow on the mental health and well-being of Cambodians, ravaged the country between 1975 and 1979, leading to the deaths of approximately two million people, and the destruction of its health and social care infrastructure [3, 4]. Mental health service infrastructures have been set up in the last thirty years to support the mental health of Cambodians but these have been shaped by very limited human resource funding [4, 5]. There is approximately 1 psychiatrist per 260,000 people compared with 1 per 9,300 people in the UK [5, 6]. There are also limited community mental health services that are provided outside of Phnom Penh (capital of Cambodia), leaving mental healthcare largely inaccessible to the 68% of the population that live in rural areas [2, 7]. Cambodians also experience a number of daily stressors including the risk of poverty, food insecurity, financial worry, intimate partner violence, and addiction which have been associated with poor mental health [8]. Social inequality and social exclusion heavily influence the development, maintenance and recurrence of common mental health issues and disorders [9, 10]. Cambodians with mental health problems are amongst the most vulnerable citizens as discrimination and religious stigma can lead to their exclusion from economic and social activities, and exercising fundamental human rights [4, 11, 12].

The negative psychological and social impact of having a mental health problem is likely to be amplified if the person is also experiencing a physical disability [4]. There is extensive evidence, which as been generated in high-income countries, that persons with physical disabilities experience much higher rates of common mental health disorders than the general population [13, 14]. In a systematic review, examining rates of anxiety and depression following limb amputation, McKechnie and John [15] highlighted the significantly higher levels of anxiety and depression experienced by this clinical population versus the general population. Cree et al. [14] conducted an analysis of 17.4 million participant’s data from the US Centers for Disease Control and Prevention, and found that persons with mobility disabilities experience distress 2.4 times more frequently than the general population. There has been limited research examining the prevalence of mental health issues and disorders amongst persons with physical disabilities in Cambodia. Maddock et al. [16] examined the rates of psychological distress and PTSD symptoms amongst (*N* = 213) prosthetic and orthotic (P&O) service users in Phnom Penh, Cambodia and found that 31.5% were experiencing mild-to-moderate psychological distress, with 13.6% reporting severe psychological distress symptoms indicative of an underlying depressive or anxiety disorder. 46% of this sample of P&O service users met the criteria for probable PTSD [16]. There are a number of psychological, social and physical factors which may help to explain the higher rates of psychological distress experienced by persons with physical disabilities, which are likely to be exacerbated in LMIC countries such as Cambodia. These include being at a higher risk of poverty [17], having to contend with consistent psychological adjustments (e.g., experiencing feelings of sadness, irritability, insecurity, anger, inferiority) [18], experiencing physical pain [19], issues in mobility [20, 21] and more limited access to healthcare [4, 22].

It has been consistently argued that policymakers in LMIC countries should facilitate training in mental health, with the aim of integrating mental health service infrastructures within primary care settings [23, 24]. This would help to close mental health treatment gaps, enhance access to mental healthcare, and generate improved mental health outcomes [24, 25]. In order to reduce common mental health disorders, in the absence of appropriate numbers of mental health professionals and funding allocations, particular attention will need to be paid to the generation of innovative and culturally appropriate mental health interventions [25]. Ensuring that mental health interventions are culturally appropriate is particularly important as the effectiveness of the importation of Western-based therapy models has been undermined by failures to fully consider levels of mental health literacy and Cambodian’s own unique cultural idioms of distress [26, 27]. A number of services applying Western-based therapy models have been criticized for being culturally insensitive, infantilizing, ethnocentric and demeaning, reducing the extent to which they can integrate within local communities, and generate both acceptance and impact in Cambodia [25, 26]. It is thus important, that the evidence base for potentially low cost, effective and culturally appropriate interventions, particularly in resource constrained countries such as Cambodia, is strengthened [24]. Evidence of effective and culturally appropriate mental health interventions of this nature is likely to support the development of evidence-based health policies and practice in Cambodia, and improve the capacity of the Cambodian government and NGOs to address the needs of their patient populations [28].

One strategy used in human resource constrained settings is to provide tailored mental health training to other professional groups who interact with patient groups on a daily basis, and allow them to administer culturally appropriate mental health support [29, 30]. This process is referred to as task-shifting, and in a systematic review of psychological and social interventions for mental health issues and disorders in Southeast Asia, Maddock et al. [31] found good quality evidence that this approach has been effective in reducing common mental health disorders when used within Southeast Asian contexts with clinical populations. Maddock et al. [31] also found promising preliminary evidence that meditation could reduce depression and anxiety symptoms of clinical populations. In another systematic review, James et al. [32], highlighted that peer support interventions (including mutual support groups), which are adapted to fit local cultures and values, are likely to be viable and effective interventions in resource constrained LMICs such as Cambodia as it is an economically viable way to engage with underserved populations at a community level. In order to reduce psychological distress amongst persons with physical disabilities in Cambodia, Best et al. [33] developed and culturally adapted a peer-led, group based, mental health intervention (named ‘Friendship’ groups) which incorporated mindfulness meditation, delivered by trained Prosthetists and Orthotists, using a task shifted approach within a clinical setting in Phnom Penh. Best et al. [33] provided some promising initial evidence of the feasibility and acceptability of Friendship groups in reducing psychological distress, PTSD symptoms and worry amongst this patient population.

There are insufficient good-quality replicated studies which have been completed to draw firm conclusions about the effectiveness of culturally appropriate interventions which can reduce depression, anxiety, and PTSD symptoms amongst clinical populations in Cambodia [31]. Replication studies are a means to confirm findings, improve processes, assess generalizability, and expand conclusions in a range of cohorts and across clinical settings [34]. Replication studies are needed to accurately advise policymakers, service commissioners, providers, and funding agencies about the optimal forms of mental health programmes to invest in, particularly those where limited resources exist [35]. Intervention studies which use controlled trial designs are also needed in order to improve the rigor for the emerging evidence base for the potential effectiveness of Friendship groups [29].

### Aims and hypotheses

This study assessed the impact of Friendship groups against a control condition. In order to improve the rigor and examine the Friendship groups ability to reproduce the empirical results found in Best et al. [33], the study looks to replicate, and expand, the findings beyond persons with physical disabilities in Phnom Penh to persons with physical disabilities across Cambodia.

More specifically, this study used a non-randomised design methodology, with an aim to:

1. Assess the effectiveness of the Friendship programme at reducing psychological distress and PTSD (primary outcomes) among an expanded cohort of prosthetic and orthotic (P&O) service users.
2. Assess the effectiveness of the Friendship programme at reducing worry, rumination, and increasing a number of mindfulness-based variables (secondary outcomes) among an expanded cohort of P&O service users.
3. Assess and confirm differences between Friendship groups and the control condition.

It is hypothesised that:

- participants in the Friendship group will report improvements in the assessed primary and secondary outcomes.
- participants in the Friendship group will report greater, significant changes in assessed outcomes when compared to the control group.
- findings will support/confirm, through replication, the efficacy of the Friendship programme among (P&O) service users in Cambodia.

## Methods

### Design

A non-randomised control design with repeated measures (pre-post design) was conducted to assess and confirm the effects of the Friendship group on psychological distress, PTSD symptoms, worry, rumination and mindfulness-based variables. This study was conducted and reported in accordance with the CONSORT guidelines [36].

### Participants

This study was embedded as part of a mental health screening and intervention programme for persons receiving prosthetic and orthotic services for the management of a physical disability. Participants were recruited from three specialist clinics in Cambodia, including one urban clinic (Phnom Penh) and two rural clinics (Kampong Som and Kampong Chhnang). Screening took place from March 2023 to June 2024 and a total of 465 P&O service users were screened. The eight-week Friendship group was designed to reduce mild to moderate psychological distress [33]. As such, inclusion criteria for this study were P&O service users over the age of 18 years of age who scored between 20 and 29 on the Kessler Psychological Distress Scale, indicating that they currently experiencing mild to moderate levels of psychological distress [37]. Exclusion criteria were P&O service users who scored below 20 on the K10, indicating that they were likely to be well, or 30 + on the K10, indicating that the person was currently experiencing severe distress, indicative of the presence of a mental disorder [38]. Persons scoring 30 + were referred for specialist mental health support.

A total of 74 patients were deemed to have met the inclusion criteria, with 53 patients consenting to be a part of the study. Due to ethical concerns, and the risk of mild to moderate psychological distress developing into more severe mental health disorders, we did not develop a randomised controlled sample, as this would have led to patients who needed support, in an already constrained resource setting, being delayed in receiving a potentially effective intervention. Following on from the learning attained from Best et al. [33] the authors anticipated a time lag between participants being initially screened and enough participants being recruited for different Friendship groups to begin. This created an opportunity to create a non-randomised control group, improving the research design and scientific rigor of the emerging evidence base examining Friendship group programmes. After their initial screening, participants who had been waiting for eight weeks had measures taken again, allowing a waitlist control to be established. The measures taken at eight weeks also functioned as the participants pre-intervention scores (*n* = 23). If a participant completed the screening and pre-intervention measures but did not proceed to the Friendship group (*n* = 16) their data also formed part of the waitlist control group data. If a Friendship group began within eight weeks of participants being screened, the participants screening score acted as their pre-intervention measures (*n* = 10). Post intervention measures were taken from all Friendship group participants (*n* = 33). All study procedures followed were in accordance with the ethical standards of the responsible committee on human experimentation (institutional and national) and with the Helsinki Declaration of 1975, as revised in 2000. Informed consent was obtained from all participants for being included in the study. Participants were not compensated for their participation in this study. Methods used in the study were approved by the National Ethics Committee for Health Research in Cambodia (#207 NEHCR). The sample demographics are highlighted in Table 1 below.

### Intervention

The development and details of the Friendship group, it’s theoretical basis, and structure have been described elsewhere [33]. Briefly, the Friendship group was developed as part of an existing international research and mental health practice collaboration between Irish, UK and Swedish mental health and physical disability academics and Cambodian mental healthcare providers, service user representative groups and mental health academics [33]. The Friendship group is an eight-week task-shifted multiple component mental health intervention programme which incorporates peer support, psycho-education, mindfulness-based practices, and behavioural activation. Friendship group sessions were delivered weekly for 1.5 h per week. Weekly sessions were facilitated by two trained Prosthetist/Orthotists from the Cambodian School of Prosthetics and Orthotics. Each weekly session followed the same delivery format: (1) group meditative exercise (10 min), (2) check in about daily and weekly activities with the facilitator identifying common themes and life challenges for discussion (60 min), (3) summary of group discussion and homework planning (10 min), and (4) final group meditative exercise and group close (10 min). The Friendship group was developed with a specific focus on incorporating Cambodian cultural idioms of distress and ameliorating issues in health literacy by empowering participants to set the agenda for the issues that were most important to them each week. The incorporation of mindfulness at the beginning and the end of each weekly session was particularly significant given its resonance with Buddhist practices prevalent in Cambodian culture [39]. Behavioral activation strategies and brief psycho-education were integrated into the sessions to promote engagement with daily activities, an approach that has proven effective in similar resource-constrained settings [40]. A manual was developed for facilitators to ensure consistency across sessions, with clear guidelines on the use of meditation, group discussions, and follow-up homework activities. This structured approach was intended to foster emotional regulation, encourage reflection, and reduce psychological distress, particularly by addressing common symptoms such as worry, anxiety, and PTSD [41, 42]. In this study, a total of four Friendship groups were delivered and only began once a group had been filled. Two friendship groups were delivered in Kampong Som and two in Kampong Chhnang. No friendship group was delivered in Phnom Penh due to low numbers of interested participants. The minimum number of participants needed for a group to begin was eight with a group maximum group capacity set at twelve participants.

### Measures

#### Psychological distress: Kessler psychological distress scale (K10) [36]

Psychological distress i.e., non-specific distress related to feelings of anxiety and depression was measured with the 10-item K-10 [36, 37]. The K-10 has been validated in Cambodia’s local language, Khmer [43] and allows patients to be screened as likely to: be well (score < 20), experiencing mild distress (score = 20–24), moderate distress (score = 25–29) or likely to have a severe distress (score ≥ 30) indicative of the presence of a mental disorder [37]. In a study with persons experiencing physical disabilities, Maddock et al. [16] reported the high level of internal consistency of the Khmer version of the K-10 (Cronbach’s Alpha 0.95). The Cronbach’s alpha for this study was 0.83.

### Primary care PTSD screen for DSM-5 (PC-PTSD-5) [42]

PTSD symptoms were measured using the PC-PTSD-5 [42]. The PC-PTSD-5 commences with a yes/no question which screens for lifetime exposure to traumatic events [42]. If the patient experiences a lifetime exposure to trauma, they are asked to answer 5 additional yes/no questions about the impact of this exposure to trauma has affected them over the last month. Scores on the PC-PTSD-5 range from 1 to 5 [42]. The PC-PTSD-5 was validated with a US veteran population in a primary care setting, and a cut off score of 3 was found to achieve optimal sensitivity for persons with PTSD. The PC-PTSD-5 was translated by a Clinical Psychologist in Cambodia (NE) with both clinical practice and research expertise in PTSD in Maddock et al. [16] and found to be internally consistent, with a Cronbach’s Alpha of 0.77. This version of the PC-PTSD-5 was also used for this study, and the Cronbach’s alpha for this study was 0.86.

### Rumination-Reflection Questionnaire-Rumination subscale-Khmer (RRQ-R-Kh) [17]

The RRQ-R-Kh is a 9-item Khmer version of the RRQ-R, which assesses the level of respondent engagement in rumination, i.e. repetitive thoughts about past experiences [44]. While the original RRQ-R comprises of 12 items [44], the RRQ-R-Kh is a 9-item version translated and culturally validated with person experiencing physical disabilities in Cambodia. Consistent with the RRQ-R, strong support has been found for reliability and validity of the RRQ-R-Kh [17]. The Cronbach’s alpha for this study was 0.81.

### The 3 item Penn state worry questionnaire (PSWQ) [45]

Worry in this study was measured by the 3-item Penn State Worry Questionnaire (PSWQ-3) [45]. The PSWQ-3 has been found to have comparable convergent, discriminant validity, and internal consistency to the 16-item PSWQ in anxiety disorder screening, and has been found to be a particularly good measure of pathological worry [45]. For this study the PSWQ-3 was translated into Khmer by the same Clinical Psychologist (NE) who translated the PC-PTSD-5. The PSWQ-3 has not been culturally validated into Khmer, however Maddock et al. [42] used the same version of the PSWQ-3 and found that it was internally consistent (Cronbach’s Alpha 0.88) in sample of persons with physical disabilities in Cambodia. The Cronbach’s alpha for this study was 0.87.

### Southampton mindfulness questionnaire-Khmer (SMQ-KH) [46]

The SMQ-Kh is 14-item translated and culturally validated measure of mindfulness [46]. The original SMQ comprises of 16 items with a single factor structure measuring mindfulness [47]. The SMQ-KH, which was translated and culturally validated with person experiencing physical disabilities in Cambodia, has a six-factor structure, and measures different facets of mindfulness including non-attachment/letting go, acceptance, non-judgment, non-reactivity, absence of aversion, and mindful observation/attention regulation skills [46]. Maddock et al. [46] found strong support for the reliability and validity of the SMQ-KH. The Cronbach’s alpha for this study was 0.67.

### Data analyses

Data were analysed using Stata 18 software [48]. The data were screened for missing values and any error cases, such as extreme outliers. There were no missing values on any of the outcomes. Repeated-measures analysis of covariance (ANCOVA) was used to assess changes in outcome measures between the Friendship group and the control. The effect size was reported based on the partial eta-squared values. Effect size interpretation was based on Cohen [49] who identified a partial *η*^2^ = 0.2 as a small effect, 0.5 = as a moderate effect, and 0.8 = as a large effect size. No *p* value adjustment was made for multiple comparisons, as controlling for Type 1 error in this manner is likely to increase the chances of Type 2 error [50]. When significant differences were found between the Friendship and control group, paired sample t-tests were used to assess within group changes from pre to post Friendship group.

## Results

**Fig. 1:**
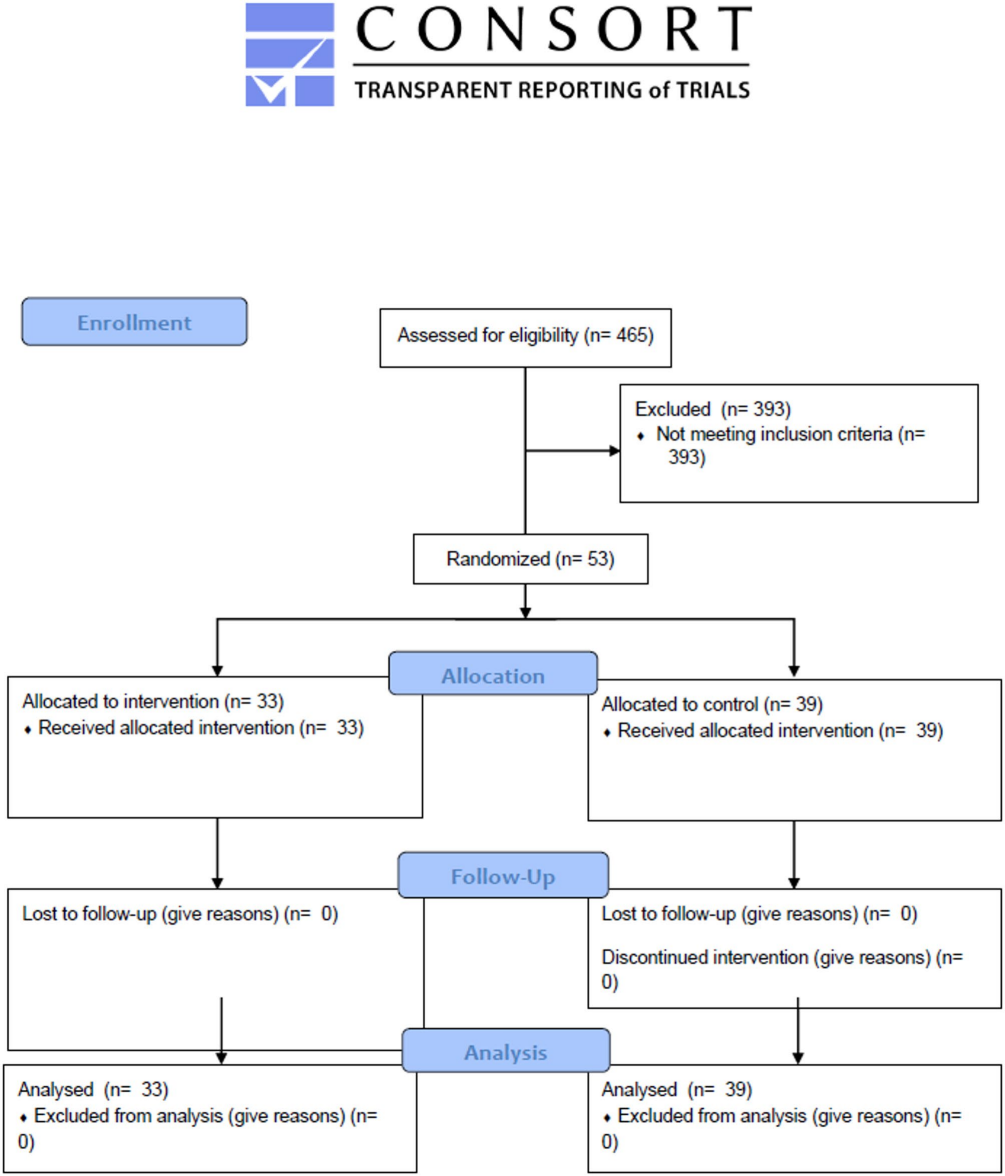
CONSORT 2010 Flow Diagram

Figure 1 illustrates the study participant flow from recruitment to completion. There were no ill/unintended negative effects, difficulties or complaints reported by the Friendship group participants. The participants demographic details are reported in Table 1.

**Table 1:** Demographics

	Friendship Group (*n* = 33)	Control (*n* = 38)
Age (25–34 years)	2	0
Age (35–44 years)	6	8
Age (45–54 years)	5	6
Age (55–63 years)	16	19
Age (64 + years)	4	5
Female n (%)	6	6
Male n (%)	27	33
Single n (%)	3	1
Married n (%)	27	33
Divorced (%)	1	2
Widowed (%)	2	3
Urban n (%)	11	11
Rural n (%)	22	28

**Table 2:** Means, standard deviations (in parentheses), and ANCOVA test statistics

	Friendship Group M(SD)	(Control) M(SD)	F	*p*	η2
**Psychological Distress**					
Pre-intervention Post-intervention	22.58 (3.26) 14.18 (5.01)	21.94 (2.35) 22.5 (3.26)	100.69	0.00	0.6
**PTSD**					
Pre-intervention Post-intervention	2.5 (0.93) 0.88 (0.83)	2.25 (1.28) 2 (1.07)	0.96	0.35	0.09
**Worry**					
Pre-intervention Post-intervention	6.67 (1.57) 5.94 (1.43)	7.11 (1.72) 6.32 (0.933)	1.13	0.29	0.016
**Rumination**					
Pre-intervention Post-intervention	33.42 (2.14) 27.88 (5.77)	31.63 (3.89) 34.02 (2.05)	33.92	0.00	0.33
**Mindful Observation/Attention Regulation**					
Pre-intervention Post-intervention	9.51 (0.82) 9.82 (1.42)	9.44 (1.7) 9.46 (0.91)	0.02	0.89	0.00
**Acceptance**					
Pre-intervention Post-intervention	14.03 (2.24) 14.45 (1.97)	13.49 (2.75) 13.38 (2.11)	1.43	0.24	0.02
**Non-reactivity**					
Pre-intervention Post-intervention	6.52 (1.3) 7.72 (2.27)	6.44 (1.46) 6.43 (1.17)	1.92	0.17	0.03
**Non-judgement**					
Pre-intervention Post-intervention	6.35 (0.74) 6.4 (1.6)	7.15 (2.05) 6.28 (1.15)	0.89	0.35	0.01
**Letting Go/Reduced Attachment**					
Pre-intervention Post-intervention	7.37 (1.56) 6.94 (1.97)	7.59 (2.05) 9.15 (1.31)	0.59	0.44	0.01
**Non-Aversion**					
Pre-intervention Post-intervention	12.57 (1.69) 14.73 (1.50)	12.97 (0.81) 12.87 (0.73)	1.33	0.25	0.02

### Differences between the friendship and control group

The means and standard deviations of the pre and post Friendship group and control group scores along with results of our ANCOVA analyses are presented in Table 2. The Friendship group participants reported moderate to large significant reductions in psychological distress from pre to post intervention compared to the control group F (1, 71) = 8.06, *p* = 0.00, η^2^ = 0.6. The Friendship group participants reported lower psychological distress score post group, when compared to pre-programme levels after participating in the Friendship group. This mean difference (MD = -8.4) was statistically significant t(32) = -10.51, *p* = 0.00. The Friendship group participants also experienced small to moderate significant reductions in rumination from pre to post intervention compared to the control group F (1, 71) = 33.92, *p* = 0.00, η^2^ = 0.33. The Friendship group participants reported lower rumination scores post group, when compared to pre-group levels. This mean difference (MD = -5.55) was statistically significant t(32) = 5.39, *p* = 0.00.

There were no significant group differences between the Friendship group and control group on PTSD symptoms F (1, 15) = 0.96, *p* = 0.35, η^2^ = 0.09, worry F (1, 70) = 1.13, *p* = 0.29, η^2^ = 0.09, non-attachment F (1, 71) = 0.08, *p* = 0.78, η^2^ = 0.01, acceptance F (1, 71) = 2.09, *p* = 0.15, η^2^ = 0.03, non-judgement F (1, 71) = 3.52, *p* = 0.07, η^2^ = 0.05, non-reactivity (1, 71) = 1.22, *p* = 0.27, η^2^ = 0.02, aversion F (1, 71) = 1.16, *p* = 0.29, η^2^ = 0.02, or mindful observation/attention regulation F (1, 71) = 0.28, *p* = 0.59, η^2^ = 0.004.

## Discussion

This non-randomised controlled study examined the effectiveness of Friendship groups at reducing psychological distress, PTSD symptoms, worry, rumination, and mindfulness-based variables. It had the added aim of replicating the results of a mixed methods study conducted by Best et al. [33] with a wider cohort of P&O service users. The significant changes found in psychological distress is consistent with the study’s hypotheses and replicate the independent results found in Best et al. [33] that Friendship group participation leads to reduced psychological distress. Our findings are also supported by Cooper et al. [51] who, in a systematic umbrella review of the effectiveness of peer support groups, found evidence that peer support groups could be an effective way to reduce depression and anxiety in clinical populations. These are encouraging findings and highlight the potential acceptability, effectiveness, and durability of Friendship groups at reducing psychological distress in P&O service users [16].

Our findings also replicate Best et al. [33] in that the Friendship group participants did experience mean reductions in PTSD symptoms, worry and increases in different facets of mindfulness. The Friendship group participants did not however experience significant changes in PTSD symptoms, worry or facets of mindfulness versus the control group. There was only a very small number of participants who reported (*n* = 8) PTSD symptoms before the Friendship group began, and this indicates that this study likely did not have sufficient power to detect between group effects. Further research is needed, with larger samples of persons with PTSD symptoms at baseline to be able to determine if the Friendship group can achieve a significant effect versus a control condition. Our study’s findings differ from Best et al. [33] in that we found significant between and within group effects of the Friendship group on rumination. The differences in this finding may be due to the different measures of rumination used to assessed rumination in both studies. Our study and Best et al. [33] used versions of rumination subscale of the Rumination reflection questionnaire [44]. Our study however used a culturally adapted and validated 9-item RRQ-R [17] which may have been more sensitive in its capacity to assess change in rumination than the translated 12-item measure used in Best et al. [33]. Best et al. [33] found a significant within group changes in worry in participants who completed the Friendship group. Using the same measurement of worry (Ch-CAMS-R), we found that though worry did reduce post Friendship group, there were no significant between group effects versus the control group.

The qualitative accounts of participants who completed the Friendship group in Best et al. [33] consistently highlighted how culturally appropriate and beneficial the 10-minute mindfulness-based exercises which took place at the start, and then at the end of each Friendship group session (20-minutes in total) were in supporting increased feelings of calmness, and the regulation of negative thoughts and challenging emotions (e.g., anger or sadness). Mindfulness is like a muscle that needs consistent exercise [39], and systematic review and meta-analysis conducted by Blanck et al. [41] highlighted how isolated mindfulness-based practices can reduce depression and anxiety symptoms. However, prediction theories of mindfulness share the idea that greater doses of mindfulness-based practices are associated with greater responses in psychological outcomes through supporting meditators to disengage from more reactive negative thinking, thereby reducing tendencies to worry and the risk experiencing psychological distress [52, 53, 54]. Though there is consistent evidence that there is a dose-response relationship between the amount of mindfulness-based practices engaged in by participants, and changes in mindfulness and facets of mindfulness, there is no clear recommendation on what the most helpful minimum dose of mindfulness is, which would likely lead to significant changes [53]. As such our results appear to indicate that though the 20 min of mindfulness-based practice per week does improve facets of mindfulness, more mindfulness-based practice per week is likely to be required to achieve significant effects. The Friendship programme contained homework which required participants to select a behavioural tasks designed to get participants to re-engage in daily activities. The addition of mindfulness-based practices e.g., 10–20 min of practice per day, and additional behavioural activation tasks to this homework, could lead to reduced worry, increased mindfulness, and further reduce the risk of participants experiencing psychological distress in further iterations of the programme [54, 55]. The addition of psycho-education on the roles that mindfulness practices and behavioural activation play in reducing worry, rumination, psychological distress and PTSD could also support improved outcomes by increasing mental health literacy and homework adherence [27, 31, 54, 55].

### Limitations and future research

This study’s findings should be considered preliminary due to its limitations. The lack of an active control group means that we cannot exclude the possibility that significant effects found on psychological distress and rumination may be due to non-Friendship group related factors such as receiving attention, or being a part of a credible support programme. The lack of an active control group also means that the improvements experienced by the Friendship group participants (who self-selected for inclusion in this study) may be due to expectations that they would improve rather than due to Friendship group participation. This study also measured all variables with self-report measures, which means that common methods bias, which may have inflated the effects found in this study cannot be ruled out [56]. While self-report measures used in the study were validated in Cambodia it is acknowledged that they were not initially developed within this context. Proponents of the emic viewpoint would suggest that this may introduce ethnocentric biases into our study and may not fully reflect local perspectives. The limited nature of the assessment and reporting of the Friendship group’s treatment fidelity limits the reliability and generalizability of this study [57, 58]. In light of this study’s results, in order to move the literature on the use of Friendship groups with P&O service users in Cambodia forward, further research using randomized controlled trial designs, examining the effectiveness of Friendship groups versus an active comparison control group are needed. Future research should also focus on the acceptability, and effectiveness of Friendship groups in reducing psychological distress in other jurisdictions. Due to the feasibility and success of this peer-led model, further research could try to replicate these outcomes in larger, more varied populations and clinical environments. It would also be important to examine the longer-term effects of Friendship groups with follow up assessments 6 and 12-months post-Friendship group participation to confirm the sustainability of its effects on psychological distress and rumination across time. Future studies might also look into incorporating more intensive mindfulness practices, psycho-education and behavioural activation activities, or extending the duration of the programme to see its longer-term effects on PTSD and other mental health outcomes [41]. In order to help to address the social determinants of mental health on P&O service users, future research on Friendship groups could explore if the addition of an interagency referral (e.g., to Habitat for Humanity) and/or educational content on identified social risk factors, such as preventing poverty by accessing cash transfers from the Cambodian government, could lead to more sustained mental health recoveries.

## Conclusion

This study is the only controlled evaluation of a culturally appropriate task shifted multiple component mental health intervention for persons with physical disabilities in Cambodia. The results from this study evidence the acceptability, effectiveness, and durability of Friendship groups at reducing psychological distress of P&O service users. These findings add to the growing literature supporting the need for culturally adapted, affordable mental health interventions in LMIC countries, where access to specialised care remains limited [31]. Though more research evaluating Friendship groups is needed, particularly in other jurisdictions, the results from this study provide reasonably clear guidance that if P&O service providers deliver Friendship groups within their services, that their service users will likely experience improved mental health outcomes. These results also point to the importance of continuing investment in scalable and culturally appropriate mental health interventions to help close treatment gaps in LMIC countries such as Cambodia [59].

## Data Availability

The datasets used and analysed during the current study are available from the corresponding author on reasonable request.
